# Low Tumor-to-Stroma Ratio Reflects Protective Role of Stroma against Prostate Cancer Progression

**DOI:** 10.3390/jpm11111088

**Published:** 2021-10-26

**Authors:** Paulina Nastały, Julia Smentoch, Marta Popęda, Emanuele Martini, Paolo Maiuri, Anna J. Żaczek, Marek Sowa, Marcin Matuszewski, Jolanta Szade, Leszek Kalinowski, Magdalena Niemira, Burkhard Brandt, Elke Eltze, Axel Semjonow, Natalia Bednarz-Knoll

**Affiliations:** 1 Laboratory of Translational Oncology, Medical University of Gdańsk, 80-210 Gdańsk, Poland; paulina.nastaly@gumed.edu.pl (P.N.); julia.smentoch@gumed.edu.pl (J.S.); marta.popeda@gmail.com (M.P.); azaczek@gumed.edu.pl (A.J.Ż.); 2 FIRC (Italian Foundation for Cancer Research), Institute of Molecular Oncology (IFOM), 20139 Milan, Italy; emanuele.martini@ifom.eu (E.M.); paolo.maiuri@ifom.eu (P.M.); 3 Department of Urology, Medical University of Gdańsk, 80-214 Gdańsk, Poland; mareczek.so@gumed.edu.pl (M.S.); marcin.matuszewski@gumed.edu.pl (M.M.); 4 Department of Pathomorphology, Medical University of Gdańsk, 80-214 Gdańsk, Poland; jszade@gumed.edu.pl; 5 Department of Medical Laboratory Diagnostics-Biobank, Medical University of Gdańsk, 80-210 Gdańsk, Poland; leszek.kalinowski@gumed.edu.pl; 6 Biobanking and Biomolecular Resources Research Infrastructure (BBMRI.pl), 80-214 Gdańsk, Poland; 7 Clinical Research Centre, Medical University of Bialystok, 15-276 Bialystok, Poland; magdalena.niemira@umb.edu.pl; 8 Institute of Clinical Chemistry, University Medical Centre Schleswig-Holstein, 24105 Kiel, Germany; burkhard.brandt@uksh.de; 9 Institute of Pathology Saarbruecken-Rastpfuhl, 66113 Saarbruecken, Germany; e.eltze@pathologie-saarbruecken.de; 10 Department of Urology, Prostate Center, University Clinic Münster, 48149 Münster, Germany; axel.semjonow@ukmuenster.de

**Keywords:** prostate cancer, tumor-to-stroma ratio, biochemical recurrence, tumor progression, digital pathology

## Abstract

Tumor-to-stroma ratio (TSR) is a prognostic factor that expresses the relative amounts of tumor and intratumoral stroma. In this study, its clinical and molecular relevance was evaluated in prostate cancer (PCa). The feasibility of automated quantification was tested in digital scans of tissue microarrays containing 128 primary tumors from 72 PCa patients stained immunohistochemically for epithelial cell adhesion molecule (EpCAM), followed by validation in a cohort of 310 primary tumors from 209 PCa patients. In order to investigate the gene expression differences between tumors with low and high TSR, we applied multigene expression analysis (nCounter^®^ PanCancer Progression Panel, NanoString) of 42 tissue samples. TSR scores were categorized into low (<1 TSR) and high (≥1 TSR). In the pilot cohort, 31 patients (43.1%) were categorized as low and 41 (56.9%) as high TSR score, whereas 48 (23.0%) patients from the validation cohort were classified as low TSR and 161 (77.0%) as high. In both cohorts, high TSR appeared to indicate the shorter time to biochemical recurrence in PCa patients (Log-rank test, *p* = 0.04 and *p* = 0.01 for the pilot and validation cohort, respectively). Additionally, in the multivariate analysis of the validation cohort, TSR predicted BR independent of other factors, i.e., pT, pN, and age (*p* = 0.04, HR 2.75, 95%CI 1.07–7.03). Our data revealed that tumors categorized into low and high TSR score show differential expression of various genes; the genes upregulated in tumors with low TSR score were mostly associated with extracellular matrix and cell adhesion regulation. Taken together, this study shows that high stroma content can play a protective role in PCa. Automatic EpCAM-based quantification of TSR might improve prognostication in personalized medicine for PCa.

## 1. Introduction

Prostate cancer (PCa) is the second of the most frequently diagnosed cancers among men worldwide [1,2]. Although the prostate-specific antigen (PSA) blood test is a widely used screening tool allowing for earlier diagnosis, it possesses some limitations in identifying patients with significant PCa or at the high risk of progression [3]. Therefore, there is a need for other prognostic marker(s) refining risk stratification and/or supporting treatment decisions.

Tumoral stroma is recognized as one of the key players [4,5] during cancer development, progression [6], or even therapy resistance [7]. Its main role includes the formation of structure and/or remodeling of the tissue [7]. In early disease stages, intratumoral stroma may even inhibit tumor progression [7], whereas, later, it frequently influences new vessel formation, inflammation, and, in turn, promotes cancer progression [8].

The intratumoral content of the stroma alone or transformed into the so-called tumor-to-stroma ratio (TSR) was reported to be of clinical significance in multiple studies. Indeed, in different solid tumors, both low and high TSR are shown to be associated with the higher risk of cancer progression and poor patients’ prognosis [9,10]. Low TSR [11,12,13] occurs as an independent prognostic factor [14], and is associated with more aggressive features of cancer cells in diverse solid malignancies including colon [15,16,17], rectal [14], gastric [10], non-small cell lung [18], ovarian [19], cervical [20], and breast cancer [21,22,23]. On the contrary, low stroma content equivalent to high TSR refers to poor prognosis in endometrial and estrogen receptor-positive breast cancer [24,25]. Although TSR has not been described yet in PCa, the clinical significance of both no/little or a high amount of stroma content (i.e., an equivalent of TSR) was shown to be associated with worse recurrence free-survival in this type of cancer [25,26,27].

Despite its clinical potential, there has not yet been implementation of TSR scoring in routine pathology. It could be due to the variety in methodology and the lack of a standardized procedure for its evaluation. TSR is one of the markers that may suffer from reproducibility issues when assessed visually due to intra- and inter-observer variability. Digitalization and automation of the TSR evaluation process might reduce this variance and facilitate standardization of TSR assessment. Indeed, due to its numerous advantages, digital pathology is rapidly evolving and gaining interest. It allows for faster, more precise, and cost-effective work, reducing the variability between pathologists’ evaluation [28,29,30]. According to the literature data, so far, the automated digital analysis of TSR has been attempted to be performed on digital images [31], using computer-aided quantification [14] or deep learning systems [32].

In PCa, the stroma content has been reported to be automatically quantified from standard hematoxylin-eosin digital scans [26,33]. Epithelial cell adhesion molecule (EpCAM) is widely expressed in PCa [34,35,36], and can differentiate between tumor and stroma cells. Thus, in the current study, TSR was assessed automatically in digitalized EpCAM-stained tumors and correlated with the clinical outcome and molecular phenotype of tumor cells in PCa patients. Additionally, using multigene expression analysis, the tumors with low and high TSR were compared at the RNA level.

## 2. Results

### 2.1. TSR Evaluation in the Pilot and Validation Cohort

The automated method recognized TSR correctly in tumors with weak (17.80%) and moderate to strong (82.20%) EpCAM expression (Supplementary Figure S1). All tumor samples from the pilot and validation study were assessed automatically followed by visual verification.

**Figure 1:**
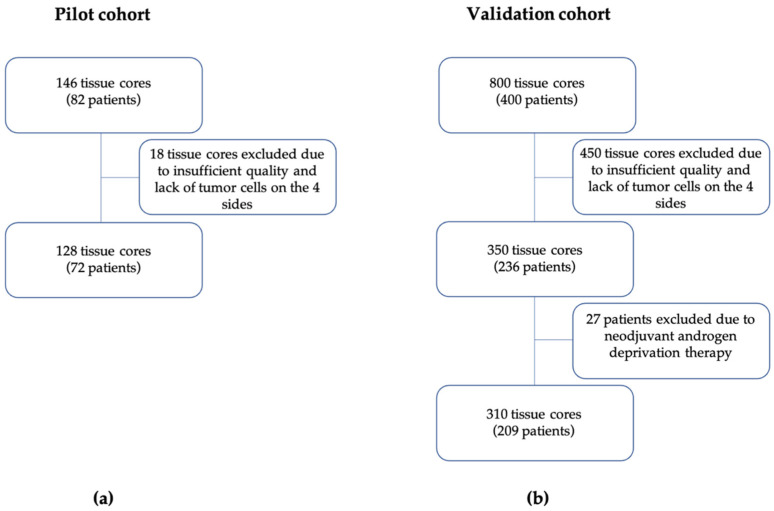
Schematic diagram of patients’ enrollment and exclusion. (**a**) Patients from the pilot. (**b**) Patients from and validation cohort.

**Figure 2:**
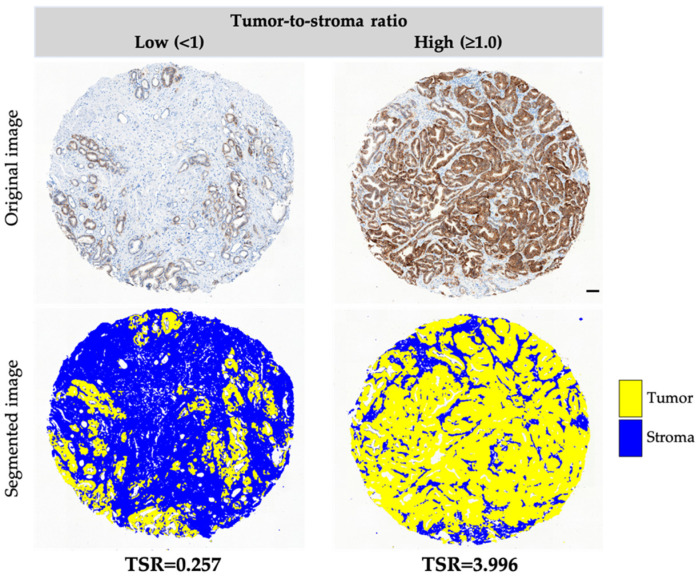
Representative photomicrographs of different TSR categories in prostate carcinomas: low and high. Tumor cells are stained with anti-EpCAM antibody and visualized in brown, whereas stroma is blue. Magnification 100×, scale bar corresponds to 100 µm.

Pilot cohort. A total of 128 tumor samples corresponding to 72 d’Amico high risk PCa patients fulfilled the technical quality and TSR assessment criteria (Figure 1A). All informative tumor samples were positive for EpCAM staining. In total, the range of TSR values detected in the informative tumor samples was 0.03 to 61.44, with the median and mean equal to 1.06 and 2.89, respectively. For each patient, the maximal value obtained from eligible tumor samples categorized as low TSR score was <1 (*n* = 31, 43.10% of all patients) or ≥1 (*n* = 41, 56.90%), respectively (Figure 2).

Validation cohort. A total of 310 tumor fragments corresponding to 209 patients fulfilled the technical quality and TSR assessment criteria, including appropriate tissue quality, presence of tumor cells visible in four sides of the specimens [17], and informative EpCAM staining. These samples were included for the further analysis (Figure 1B). In total, the range of TSR values detected in the informative tumor samples was 0.16 to 27.50, with the median and mean determined as 2.14 and 3.52, respectively. For each patient, the maximal value obtained from eligible tumor samples was categorized as low or high TSR if TSR score was <1 (*n* = 48, 23.00% of all patients) or ≥1 (*n* = 161, 77.00%), respectively (Figure 2).

### 2.2. Clinical Significance of TSR in the Pilot Cohort

**Figure 3:**
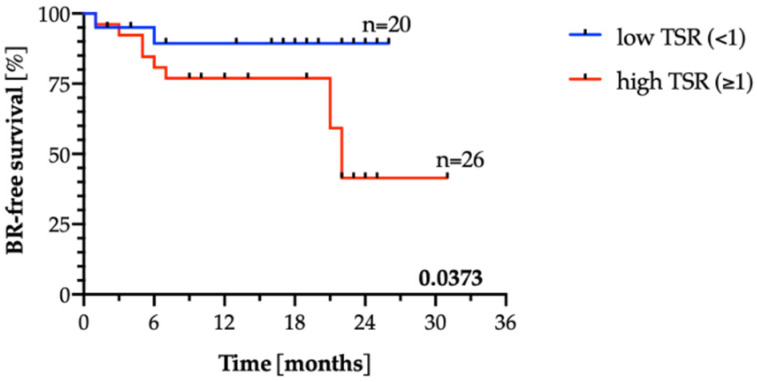
Kaplan–Meier estimates of time to biochemical recurrence (BR) in the pilot cohort (*n* = 72). Note that not all patients are included in Kaplan–Meier analysis as the exact timepoints of BR occurrence for some patients were missing.

When compared to the available clinico-pathological parameters, TSR score did not correlate to patient’s age, pT status, pN status, and preoperative PSA in the pilot cohort (Supplementary Table S1). TSR correlated to higher Gleason Grading score (Fisher’s exact test, *p* = 0.03). In the survival analysis, high TSR appeared to indicate a shorter time to biochemical recurrence in the pilot cohort of PCa patients (Log-rank test, *n* = 72, *p* = 0.0373, Figure 3). In uni- and multivariate analysis of the pilot cohort (*n* = 72) for predicting BR, including pT and pN status, age, Gleason score, and total serum PSA concentration, TSR did not show a prognostic relevance (Cox regression model, Supplementary Figure S2).

### 2.3. Clinical Significance of TSR in the Validation Cohort

**Figure 4:**
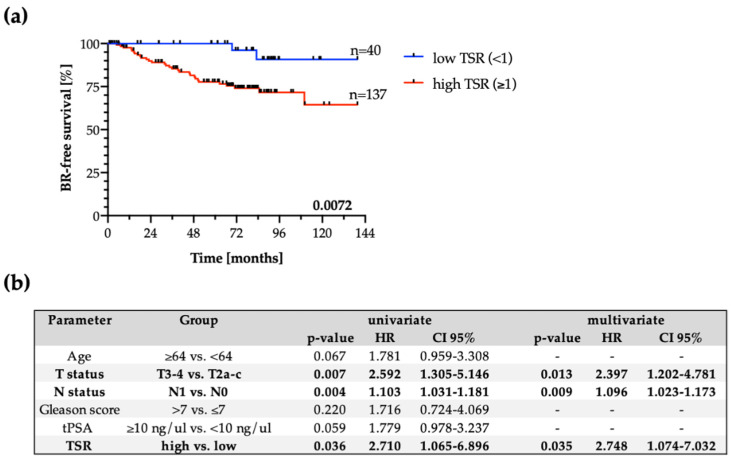
Clinical significance of TSR in a validation cohort. (**a**) Kaplan–Meier estimates of time to biochemical recurrence (BR) in validation cohort, *n* = 209. Note that not all patients are included in Kaplan–Meier analysis as the timepoints of BR occurrence for some patients were missing. (**b**) Uni- and multivariate analysis in the whole validation cohort. Statistically significant results are bolded.

TSR status did not correlate to any clinico-pathological parameter including age, pT status, pN status, Gleason Grading score, and preoperative PSA in the validation cohort (Supplementary Table S2). High TSR appeared to indicate a shorter time to biochemical recurrence in the validation cohort of PCa patients (Log-rank test, *n* = 209, *p* = 0.0072, Figure 4A). In the multivariate analysis, including pT and pN status, age, Gleason score, total serum PSA concentration, and TSR (Cox regression model), TSR predicted BR independent of other factors, i.e., pT, pN, and age (*p* = 0.04, HR 2.75, 95%CI 1.07–7.03; Figure 4B). Additionally, when the validation cohort was only limited to d’Amico high risk patients, high TSR still appeared to indicate a shorter time to biochemical recurrence (Log-rank test, *n* = 158, *p* = 0.0149, Supplementary Figure S3).

### 2.4. RNA Expression Characteristics in Relation to the TSR Score

**Figure 5:**
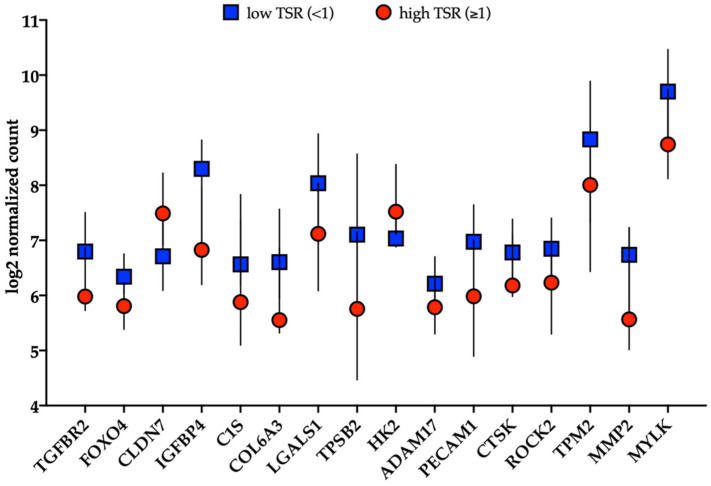
Genes diversly expressed in relation to the TSR score. Statistical significance was considered at *p*-value ≤ 0.05, *n* = 42.

In order to investigate the gene expression differences between tumors with low and high TSR, we applied multigene expression analysis (using nCounter^®^ PanCancer Progression Panel, NanoString) of 42 tissue cores, among which 21 (50%) were classified as low and 21 (50%) as high TSR. We observed that tumors with a low TSR score showed the upregulation of 14 genes and the downregulation of 2 genes (*p*-value ≤ 0.05), as shown in Supplementary Table S3. The genes upregulated in tumors with low TSR were mostly associated with extracellular matrix organization and regulation (i.e., COL6A1, TGFBR2, PECAM1, MMP2, CTSK) and cell adhesion regulation (i.e., ADAM17). Tumors categorized with low TSR score also showed higher expression of transcription factor, FOXO4, whereas 2 upregulated genes in high TSR samples were encoding hexokinase 2 (HK2, an enzyme involved in glucose metabolism) and Claudin-7 (CLDN7, a protein involved in tight junction formation), as presented in Figure 5.

## 3. Discussion

Tumor-to-stroma ratio is a simple and reliable parameter, which seems to facilitate patient’s diagnosis and prognosis in many types of solid tumors [16]. Its evaluation has the potential to be partially or fully automated. Here, we show that automatically assessed TSR can be of clinical significance in PCa patients.

In our study, we divided cohorts into low and high TSR, as in the vast majority of studies, where 50% threshold was used to categorize patients [10,11,12,16,17,29,37]. Thus, our evaluation was performed on all informative tumor samples available for each patient, and both versions of selection of minimal or maximal TSR per patient (i.e., the lowest or the highest TSR score assigned to a patient) were tested statistically (data not shown). In the presented study, high TSR showed an unfavorable prognosis. It was also shown to be an independent prognostic factor in the multivariate analysis, independent of age, pT, and pN in the validation cohort. Its lack of prognostic relevance in uni- and multivariate analysis in the pilot cohort can be biased by the low number of cases.

This observation was supported by transcriptomic analysis. Tumors categorized into low and high TSR score showed differential expression of various genes. The genes upregulated in tumors with low TSR score were mostly associated with extracellular matrix and cell adhesion regulation. Interestingly, tumors with low TSR also showed higher expression of a transcription factor FOXO4, which has been reported as a metastasis suppressor [38] in PCa. This suggests that stroma plays a protective role in PCa development. In the future, it could be worth combining the evaluation of TSR with other molecular markers. Such combination of analyzing the TSR and staining for another transcription factor (SMAD4) improved prediction of clinical outcome [39] in colon carcinoma.

Using automated digital images-based evaluation of EpCAM-stained tumor specimens, we correctly analyzed all tumor fragments of the pilot cohort and 91% of tumor fragments in the validation cohort. Automation may reduce inter-observer variation, improving the compliance and accuracy of the results, as the human eye is not able to distinguish precisely, e.g., the blank spaces caused by gland architecture in prostate tissue [14]. However, it still merits some limited assistance and visual verification. Such inaccuracies were, however, observed in only 4% of all analyzed tumors; therefore, we decided to assess them visually in order to not bias the clinical results by simple sample exclusion. In our study, tumor cells were stained with anti-EpCAM antibody (brown), which contrasted to EpCAM-negative stroma (blue). This marker-dependent protocol provides a clear and precise detection of tumor cells area in comparison to evaluation based on the standard hematoxylin-eosin staining [9,12,14,17,18,23,24,37,40,41,42,43]. Both approaches have some limitations. EpCAM is not an ideal marker to identify tumor area, as it might be downregulated in some tumor cells undergoing epithelial-mesenchymal transition [44,45]. Indeed, a few tumor fragments were EpCAM-negative and required additional visual verification. In the future, different markers of tumor cells (e.g., a cocktail of different antibodies) should be used to potentially identify all tumor cells.

The presented data suggest that, although TSR evaluation has not yet been implemented in routine pathologic diagnostics, it has the potential for integration together with the TNM staging system [15]. In PCa, TSR seems to be a simple marker to assess with high prognostic potential. Of note, the partial or even full automation of TSR evaluation is probably attainable in the broad spectrum of solid tumors. Nevertheless, further improvement and validation of the proposed methodology on a larger cohort of patients is still required.

## 4. Material and Methods

The study has been approved by the local Ethics Committee (i.e., Ethik Kommission der Aerztekammer Westfalen-Lippe und der Medizinischen Fakultaet der Westfaelischen Wilhelms-Universitaet Muenster, Germany, no 2007–467–f–S and Independent Bioethics Committee for Scientific Research at Medical University of Gdańsk, no. NKBBN/286/2018).

### 4.1. Pilot Study to Test Feasibility of Quantification Method

In a pilot study, a total of 128 primary tumor samples corresponding to 72 d’Amico high risk PCa patients, who underwent radical prostatectomy (RP) at the Department of Urology at the Medical University of Gdańsk (Poland) in years 2018–2020, were analyzed (Table 1). Tumor specimens were prepared as tumor microarrays (TMAs) that consisted of 3 tumor fragments per patient, including fragments of tumoral invasive front, and tumor with highest and dominant Gleason score. For further analysis, only tumor fragments containing fragments with the highest and dominant Gleason were included.

### 4.2. Patient Characteristics of Validation Cohort

A total of 800 tumor fragments were collected from 400 PCa patients undergoing RP at the Department of Urology at the University Clinic Münster (Germany) in the years 2002–2004. Tumor specimens were prepared as TMAs as described [46,47], wherein two fragments of a tumor with the highest Gleason score (GS) were collected from each patient. Samples were obtained from 2 tumor foci (multifocal PCa) or 2 different areas of a tumor (monofocal PCa). The last follow-up was performed in September 2019 (resulting in up to 17 years follow-up). Biochemical recurrence was defined if PSA level increased above 0.1 ng/mL after RP in two consecutive assessments. Time to biochemical recurrence was defined as time between RP and the first timepoint of PSA above 0.1 ng/mL. Patients were classified using the d’Amico classification as previously reported [48,49]. Patients that received neoadjuvant androgen deprivation therapy were excluded from the study (*n* = 27). Patients enrolled in this study were characterized by variable clinico-pathological parameters (Table 1).

### 4.3. Immunohistochemistry for EpCAM and Different Markers Associated with Tumor Cells

Tumor samples were stained immunohistochemically for epithelial cell adhesion molecule (EpCAM) in order to visualize tumor cells [50]. Briefly, tissue microarrays (TMAs) were stained with the use of anti-EpCAM, NCL-ESA Novocastra antibody (diluted 1:75), envisioned with EnVision Kit Rabbit/Mouse (Dako), and counterstained with hematoxylin. In this system, 3,3′-Diaminobenzidine (DAB) is oxidized to brown precipitate by hydrogen peroxide in a reaction catalyzed by horseradish peroxidase. Different tumor cell markers (including different keratins, E-cadherin, N-cadherin, vimentin, Ki-67, ALDH1, and others) were also stained by immunohistochemistry, evaluated, and categorized as negative or positive as described [46,47,50,51,52,53,54].

### 4.4. Evaluation of TSR

In the pilot cohort, photomicrographs of tumor fragments were acquired using the Pannoramic 250 II Flash (3D Histech, Hungary) equipped with the 20× objective. Photomicrographs of each tumor sample in the validation cohort were acquired with the transmitted light microscope Olympus BX63 Olympus (Japan) equipped with 10x objective (UPlanSApo, NA 0.4) and a 36-bit color camera DFC450C (Leica, Germany). Automated evaluation of TSR in each examined tumor sample was carried out using custom-built Jython Fiji plugin [55]. The plugin performed a color deconvolution from the RGB image to H-DAB staining using the Fiji Plugin Color Deconvolution [56]. The tumor area was recognized as the (DAB) channel of the H-DAB deconvoluted image using the Moment’s Threshold method, after median filtering. The stroma area was recognized using the hematoxylin channel of the H-DAB deconvoluted image using the Otsu’s Threshold method, after median filtering. The plugin considered valid stroma regions only the ones not overlapping with the tumor region. Then, TSR ratio was evaluated as the ratio between the area of the tumor cells and the area of the stroma. In this study, all tumor fragments were analyzed automatically and verified visually. Tumor specimens technically invalid or without tumor cells, as well as specimens without tumor cells in four sides of the field of view (north, south, east, west), according to van Pelt et al.’s recommendations [16,17], were excluded. Visual analysis of TSR was applied for EpCAM-negative tumors (*n* = 19). TSR evaluation was assessed for two tumor fragments (i.e., TMA tissue cores) per patient. Minimal and maximal value was counted for each patient (if two tissue cores were valid) in order to test their potential clinical significance. If one tumor fragment was excluded, only the remaining one was assigned to a patient. Various cut-offs (e.g., mean, median, and quartiles) were used to categorize TSR and examine its significance in PCa.

### 4.5. RNA Extraction

Total mRNA was extracted from 42 samples prepared as formalin-fixed paraffin-embedded (FFPE) primary prostate tumor tissue cores (three 20 µm-thick, unstained FFPE sections per patient) using the RNeasy Mini Kit (Qiagen, Hilden, Germany) according to the manufacturer’s protocol. RNA integrity was assessed using the Agilent 2100 Bioanalyzer (Agilent Technologies, Santa Clara, CA, USA) with the Agilent RNA 6000 Pico Kit (Agilent Technologies).

### 4.6. nCounter Gene Expression Assay

Extracted RNA (4 µL) was pre-amplified using the nCounter Low RNA Input Kit (NanoString Technologies, Seattle, WA, USA) with the dedicated Primer Pool covering the sequences of 730 immune-related genes included in the nCounter Cancer Progression Profiling Panel (NanoString Technologies). Pre-amplified samples were analyzed using the NanoString nCounter Analysis System (NanoString Technologies) according to the manufacturer’s procedures for hybridization, detection, and scanning. The collected RNA expression results were then correlated to the TSR score of the corresponding tissue core.

### 4.7. Statistical Analysis

The cut-off with the best clinical relevance was selected and presented in this study; for each patient, the maximal value obtained from informative and eligible tumor samples was categorized as low TSR (TSR score < 1), or high TSR (TSR score ≥ 1). Statistical analysis was performed using IBM SPSS software Statistics 25.0.0.2 licensed for the University of Gdańsk. Graphs were prepared using the GraphPad Prism software (8.0d licensed for the Medical University of Gdańsk). The comparison of TSR and clinical data was performed using the Chi-squared or Fisher’s exact tests. Survival analyses were performed using the Log-rank (Mantel Cox) test and visualized by Kaplan–Meier plots. Uni- and multivariate analysis was assessed using the Cox Regression model. The results were considered statistically significant at *p* < 0.05.

## 5. Conclusions

To conclude, this study indicated that automatically quantified TSR may support identification of patients at the higher risk of PCa recurrence. Thus, this methodology carries potential to further develop and improve pathological expertise and routine diagnostics in PCa, and plausibly also other solid tumors.

## Figures and Tables

**Table 1:** Distribution of clinical parameters in the pilot and validation cohort of eligible patients. PSA, prostate-specific antigen. Note that not all numbers sum up to 72 and 209 due to the missing data.

Clinical and Pathological Parameters	Pilot Cohort			Validation Cohort	
	Status	*n*	%	*n*	%
Age (years)	<median (65)	37	52.1	102	48.8
	≥median (65)	34	47.9	107	51.2
	Total	72		209	
T status	pT2	34	52.2	95	45.4
	pT3	31	47.5	94	45.0
	pT4	0	0.0	20	9.6
	Total	65		209	
N status	pN0	62	93.9	191	94.1
	pN1-2	4	6.1	12	5.9
	Total	66		203	
Gleason score sum	<7	0	0.00	52	24.9
	7	45	64.3	140	67.0
	>7	25	35.7	17	8.1
	Total	70		209	
Preoperative PSA	<10 ng/ml	46	63.9	132	65.5
	≥10 ng/ml	26	36.1	76	36.5
	Total	72		205	
d’Amico scale	low risk	0	0.0	2	1.0
	intermediate risk	0	0.0	46	22.3
	high/very high risk	72	100.0	158	76.7
	Total	72		206	
Biochemical recurrence	No	34	70.8	166	79.4
	Yes	14	29.2	43	20.6
	Total	48		209	

## Data Availability

The data presented in this study are available on request from the corresponding author.

## Supplementary Materials

The following are available online at https://www.mdpi.com/article/10.3390/jpm11111088/s1, Figure S1: Comparison of visual and automated TSR evaluation in relation to EpCAM staining intensity, Figure S2: Uni- and multivariate analysis in the whole pilot cohort (*n* = 72). Figure S3: Kaplan-Meier estimates of time to biochemical recurrence (BR) in d’Amico high risk patients from validation cohort (*n* = 158), Table S1: title Comparison of TSR status to the clinic-pathological parameters in the pilot cohort, Table S2 Comparison of TSR status to the clinic-pathological parameters in the validation cohort, Table S3 RNA expression data in tissue cores classified as low and high TSR.
