# Supplementary material for: Low Tumor-to-Stroma Ratio Reflects Protective Role of Stroma against Prostate Cancer Progression
Source: J Pers Med. 2021 Oct 26;11(11):1088. doi: 10.3390/jpm11111088 (PMC8622253; doi:10.3390/jpm11111088)
Supplement: Supplementary file 1 [file jpm-11-01088-s001.zip › jpm-1396030-supplementary.pdf]

Supplementary Materials

Table S1 Comparison of TSR status to the clinic-pathological parameters in the pilot cohort. (F), Fisher's exact test; PSA, prostate-specific antigen. Note that not all numbers sum up to 72 due to the missing data.

Variable	Informative for TSR Patients, n (%)	TSR low Patients, n (%)	TSR high Patients, n (%)
Age			
<65	37 (52.1%)	16 (53.3%)	21 (51.2%)
≥65	34 (47.9%)	14 (46.7%)	20 (48.8%)
total	71	p=0.860	
T status			
T2	34 (52.3%)	17 (63.0%)	17 (44.7%)
T3	31 (47.7%)	10 (37%)	21 (55.2%)
T4	0 (0%)	0 (0%)	0 (0%)
total	65	p=0.208 (F)	
N status			
N0	61 (93.8%)	25 (92.6%)	36 (94.7%)
N1-N2	4 (6.2%)	2 (7.4%)	2 (5.3%)
total	64	p=1.00	
Gleason Grading Score			
<7	0	0	0
7	46 (69.7%)	23 (85.2%)	23 (59.0%)
>7	20 (30.3%)	4 (14.8%)	16 (41.0%)
total	66	p=0.030 (F)	
Preoperative PSA			
<10 ng/ml	46 (63.9%)	21 (67.7%)	25 (61.0%)
≥10 ng/ml	26 (36.1%)	10 (32.3%)	16 (39.0%)
total	72	p=0.554	

Table S2 Comparison of TSR status to the clinic-pathological parameters in the validation cohort. (F), Fisher's exact test; PSA, prostate-specific antigen. Note that not all numbers sum up to 209 due to the missing data.

Variable	Informative for TSR Patients, n (%)	TSR low Patients, n (%)	TSR high Patients, n (%)
Age			
<64	102 (48.8%)	25 (52.1%)	77 (47.8%)
≥64	107 (51.2%)	23 (47.9%)	84 (52.2%)
total	209	p=0.605	
T status			
T2	92 (44.1%)	24 (50.0%)	71 (44.1%)
T3	94 (45.0%)	20 (41.7%)	74 (46.0%)
T4	18 (8.6%)	4 (8.3%)	16 (9.9%)
total	204	p=0.766	
N status			
N0	191 (91.4%)	44 (95.7%)	147 (93.0%)
N1-N2	12 (6.2%)	2 (4.3%)	11 (7.0%)
total	204	p=0.737 (F)	
Gleason Grading Score			
<7	52 (24.9%)	15 (31.3%)	37 (23.0%)
7	140 (67.0%)	32 (66.7%)	108 (67.1%)
>7	17 (8.1%)	1 (2.1%)	16 (9.9%)
total	209	p=0.148	
Preoperative PSA			
<10 ng/ml	132 (63.5%)	34 (72.3%)	98 (60.9%)
≥10 ng/ml	76 (36.5%)	13 (27.7%)	63 (39.1%)
total	208	p=0.151	
Death			
alive	195 (93.3%)	45 (93.8%)	150 (96.8%)
PCa-related	2 (1.0%)	1 (2.1%)	1 (0.6%)
not Pca-related	6 (2.9%)	2 (4.2%)	4 (2.6%)
unknown	0	0	0
total	203	p=0.572	

Table S3 RNA expression data in tissue cores classified as low and high TSR.

gene symbol	low TSR (<1) [median normalized count]	high TSR (>1) [median normalized count]	log2FC [high vs. low TSR]	p-value [Mann-Whitney-Wilcoxon test]
TGFBR2	110	62	-0.83	0.009
FOXO4	80	55	-0.54	0.010
CLDN7	104	179	0.79	0.018
IGFBP4	314	113	-1.48	0.021
C1S	94	58	-0.69	0.022
COL6A3	96	46	-1.07	0.029
LGALS1	262	138	-0.92	0.031
TPSB2	137	53	-1.36	0.031
HK2	130	183	0.49	0.034
ADAM17	73	54	-0.43	0.036
PECAM1	125	62	-1.00	0.038
CTSK	109	72	-0.61	0.043
ROCK2	114	74	-0.62	0.043
TPM2	455	256	-0.83	0.046
MYLK	831	427	-0.96	0.049
MMP2	106	46	-1.19	0.049

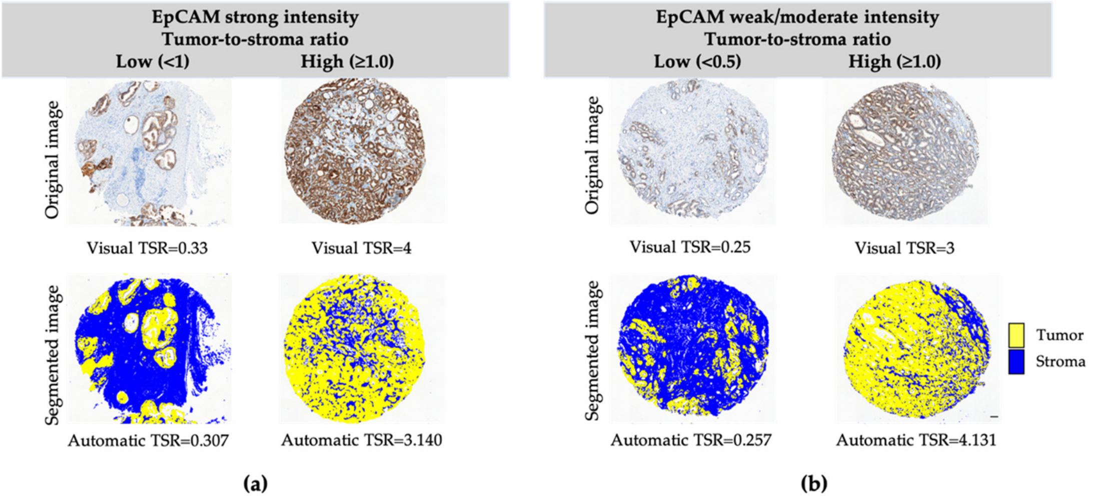

Figure S1. Comparison of visual and automated TSR evaluation in relation to EpCAM staining intensity. A, Brightfield images of tumor cells stained for EpCAM with strong staining intensity (brown) and counterstained with hematoxylin with TSR scores assessed visually (upper panel) and corresponding images of tissue cores segmented images after automated TSR analysis where tumor cells are color-coded as yellow, whereas stroma is represented by blue color (bottom panel). **B**, Brightfield images of tumor cells stained for EpCAM with weak/moderate staining intensity (brown) and counterstained with hematoxylin with TSR scores assessed visually (upper panel) and corresponding images of tissue cores segmented images after automated TSR analysis where tumor cells are color-coded as yellow, whereas stroma is represented by blue color (bottom panel). Magnification 100x, scale bar corresponds to 100 µm).

Parameter	Group	univariate			multivariate		
		p-value	HR	CI 95%	p-value	HR	CI 95%
Age	≥65 vs. <65	0.624	0.767	0.266-2.214	-	-	-
T status	T3-4 vs. T2a-c	0.032	3.571	1.118-11.411	0.075	2.977	0.895-9.897
**N status**	**N1 vs. N0**	**0.001**	**55.373**	**4.890-626.981**	**0.004**	**34.954**	**3.015-405.238**
Gleason score	>7 vs. ≤7	0.055	2.800	0.980-7.995	-	-	-
tPSA	≥10 ng/ul vs. <10 ng/ul	0.307	1.785	0.587-5.428	-	-	-
TSR	high vs. low	0.063	4.147	0.927-18.556	-	-	-

Figure S2. Uni- and multivariate analysis in the whole pilot cohort (n=72). Statistically significant results are bolded.

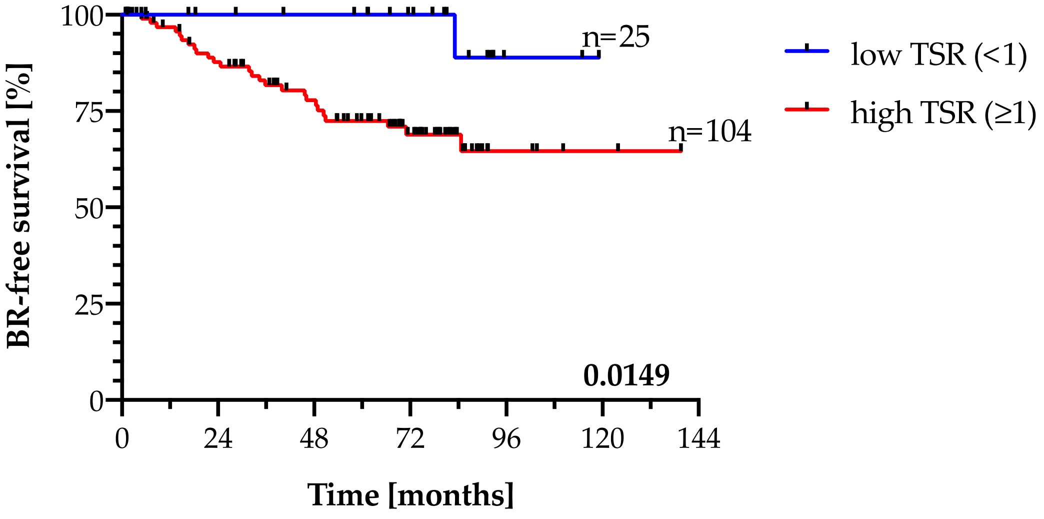

Figure S3. Kaplan-Meier estimates of time to biochemical recurrence (BR) in d'Amico high risk patients from validation cohort (n=158). Note that not all patients are included in Kaplan-Meier analysis as the timepoints of BR occurrence for some patients were unknown.
